# Beat Synchronization across the Lifespan: Intersection of Development and Musical Experience

**DOI:** 10.1371/journal.pone.0128839

**Published:** 2015-06-24

**Authors:** Elaine C. Thompson, Travis White-Schwoch, Adam Tierney, Nina Kraus

**Affiliations:** 1 Auditory Neuroscience Laboratory, Northwestern University, Evanston, Illinois, United States of America; 2 Department of Communication Sciences and Disorders, Northwestern University, Evanston, Illinois, United States of America; 3 Institute for Neuroscience, Northwestern University, Evanston, Illinois, United States of America; 4 Department of Neurobiology & Physiology, Northwestern University, Evanston, Illinois, United States of America; 5 Department of Otolaryngology, Northwestern University, Chicago, Illinois, United States of America; UNLV, UNITED STATES

## Abstract

Rhythmic entrainment, or beat synchronization, provides an opportunity to understand how multiple systems operate together to integrate sensory-motor information. Also, synchronization is an essential component of musical performance that may be enhanced through musical training. Investigations of rhythmic entrainment have revealed a developmental trajectory across the lifespan, showing synchronization improves with age and musical experience. Here, we explore the development and maintenance of synchronization in childhood through older adulthood in a large cohort of participants (N = 145), and also ask how it may be altered by musical experience. We employed a uniform assessment of beat synchronization for all participants and compared performance developmentally and between individuals with and without musical experience. We show that the ability to consistently tap along to a beat improves with age into adulthood, yet in older adulthood tapping performance becomes more variable. Also, from childhood into young adulthood, individuals are able to tap increasingly close to the beat (i.e., asynchronies decline with age), however, this trend reverses from younger into older adulthood. There is a positive association between proportion of life spent playing music and tapping performance, which suggests a link between musical experience and auditory-motor integration. These results are broadly consistent with previous investigations into the development of beat synchronization across the lifespan, and thus complement existing studies and present new insights offered by a different, large cross-sectional sample.

## Introduction

Spontaneous movement to a rhythmic beat, whether it be foot tapping to music or dancing, is a natural human behavior [1–5]. For instance, infants are predisposed to rhythmic categorization and movement [6–8], and beat synchronization is an essential component of music performance and perception across cultures [9–11]. Rhythmic entrainment, though a simple task to perform, is rich and complex in its neurobiological mechanisms, recruiting and integrating multiple neural systems such as auditory, motor, and executive centers [12,13].

The ability to tap consistently to a beat emerges in early childhood [14,15] and improves with age into adulthood [16,17]. However, in older adulthood, rhythmic entrainment becomes more variable [16–18]. Several cross-sectional studies have investigated the effect of age on rhythmic entrainment with both large sample sizes and a continuous distribution of age across a wide range [14,16–18]. For example, McAuley and colleagues (2006) investigated perceptual-motor timing tasks in 305 individuals, ranging from age 4 to age 95, and found different developmental profiles for the age groups; Drewing and colleagues (2006) tested the synchronization ability of 286 participants, ages 6–88 years, and found that performance improves in childhood and is relatively stable until old age. These studies provide evidence indicating across the lifespan, sensorimotor integration changes with age, and furthermore, in older age, synchronization may be a unique window to understand how sensorimotor integration slows.

Beyond natural development, life experiences may fine-tune rhythmic entrainment. Previous work has established a link between musical practice and greater timing accuracy in rhythmic tasks, such as beat tracking [19]; one study showed that children and adults with music training had more proficient tapping synchronization and tempo discrimination (i.e. attunement) than individuals without music training [16]. Elementary school children with one year of musical training have more accurate performance on sensorimotor rhythmic tasks than peers with no music training [20]. In fact, musical training is thought to engender benefits for a wide array of auditory-perceptual and cognitive functions throughout the lifespan [21–23]; but see [24]. This suggests that musical training—which incorporates memorizing, interacting with, and attending to sound—may be associated with advantages in auditory attention and temporal acuity, abilities which are linked to synchronization [25]. Moreover, beat synchronization performance correlates with auditory neural synchrony [26], which may be strengthened in individuals with a minimal amount of musical training early in life [27]. One should be cautious, however, that these putative musician enhancements may be due to preexisting differences, such as rhythmic competence, that draw certain individuals to pursue music training.

Relatively little is known about the impact of musical experience on beat synchronization across the lifespan, especially within a large group of subjects of various ages. Moreover, many studies of musical training have adopted stringent criteria for “musicians,” many of which are often formally trained professionals [5], as opposed to the more common case of an individual with just a few years of musical experience. We investigated beat synchronization through a uniform assessment of tapping in children, adolescents, young adults, middle-age adults, and older adults, with respect to age and musical experience. We predicted that synchronization improves into adulthood but becomes more variable in older adulthood, and that musical experience tracks with greater synchronization abilities throughout life.

## Materials and Methods

### Ethics statement

The Institutional Review Board at Northwestern University approved all experimental procedures. Written consent was obtained at the onset of participation. For the children and adolescents, written consent was obtained on behalf of the minors by parents or guardians. Also, written assent was obtained by the minors. All consent procedures were approved by the Northwestern IRB and successful acquisition of consent was documented.

### Participants

145 individuals were recruited from the Chicago area. Participants were grouped by age: children (N = 27, age range: 8.0 to 13.99), adolescents (N = 32, age range: 14.00 to 17.99), young adults (N = 18, age range: 18.0 to 21.99), middle age adults (N = 25, age range: 22.0 to 42.99), and older adults (N = 43, age range: 51.0 to 79.99) (refer to Table 1 *)*. Groups were balanced for sex, and no participant had a history of a neurologic or motor disorder. All participants were given identical instructions for the beat synchronization task (see below). Participants were monetarily compensated for their time.

**Table 1:** Subject Age Group Information.

Age Group	N	Mean Age +/- SD	Age Range	Sex
Children	27	11.04 +/- 1.72	8.0 to 13.99	15 female
Adolescents	32	16.38 +/- .78	14.0 to 17.99	17 female
Young Adults	18	20.25 +/- 1.15	18.0 to 21.99	8 female
Middle Age Adults	25	28.49 +/- 5.40	22.0 to 42.99	12 female
Older Adults	43	63.67 +/- 5.46	51.0 to 79.99	20 female

To analyze an association between musical experience and tapping performance, we conducted two analyses of musical experience, treating it as both a categorical and as a continuous variable. Each participant reported their music training history and was categorized as having “musical experience” (children: N = 16; adolescents: N = 18; young adults: N = 14; middle age adults: N = 18) or “no musical experience” (children: N = 11; adolescents: N = 14; young adults: N = 4; middle age adults: N = 7) (refer to Table 2). A liberal criterion for musical experience was adopted for this study given the robust findings of Slater et al., (2013), whereby children who underwent one year of music training tapped less variably than control children. Here, individuals with a minimum of 3 years of practicing music qualified as having musical experience. As the majority of individuals have some interaction with music at some point in their life, we restricted the “no musical experience” group to individuals with a negligible amount of musical experience (i.e., no more than 6 months of consistent practice). The older adults did not report their musical training history, and thus the older adult cohort was excluded from the analyses of effects of musical training. To treat musical experience as a continuous variable, and to ask whether the extent of musical experience is associated with beat synchronization, we calculated proportion of life spent playing music by dividing years of musical experience by years of life (thereby avoiding its colinearity with age, R(64) = .643, *p* < .001). Two children were excluded from the correlation analysis for not reporting exact years of music training.

**Table 2:** Musical Experience Categorization Information.

Age Group	No Musical Experience (N)	Musical Experience (N)
Children	11	16
Adolescents	14	18
Young Adults	4	14
Middle Age Adults	7	18
Older Adults	N/A	N/A

### IQ

For each age group, one of two non-verbal IQ tests was administered: either the matrix reasoning subtest of the Wechsler Abbreviated Scale of Intelligence (WASI [28], or the Test of Nonverbal Intelligence (TONI [29]). Thus, to compare the groups on IQ, non-verbal IQ percentiles were calculated for each subject. Though the children, young adults, middle age adults and older adults did not differ on IQ, the adolescents differed from each age group (p < .001). IQ was therefore included as a covariate in all analyses.

### Paced synchronization task

**Fig 1:**
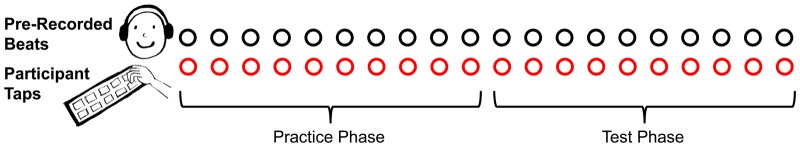
Paced Tapping Schematic. The participants practiced tapping to the sound for 20 beats (practice phase), and then immediately tapped to the sound for 20 more beats during which time their taps were recorded (test phase). The participants were asked to tap along to the beat throughout the entire task such that their taps occurred at the exact same time as the drum sounds.

We used a simple paced finger-tapping task that has been used by many laboratories to measure rhythmic entrainment [2,5,26,30,31] (see Fig 1 for a schematic). In this test, the participant heard a prerecorded, isochronously repeated snare drum sound and was asked to tap along to the beat on a NanoPad2 tapping pad (Korg, Tokyo, Japan). The participants practiced tapping to the sound for 20 beats (practice phase), and then immediately tapped to the sound for 20 more beats during which time their taps were recorded (test phase). The participants were asked to tap along to the beat throughout the entire task such that their taps occurred at the exact same time as the drum sounds; thus, this condition assesses the extent to which participants can maintain their tapping to follow the pacing stimulus. Two rates were presented for the task: 500 ms (fast) and 667 ms (slow) inter-stimulus-interval (ISI) tempos.

#### Measures of paced synchronization: variability and asynchrony

Custom-written software was used to analyze tapping variability and asynchrony (defined and described in Table 3). Variability was calculated by computing the standard deviation of the inter-tap intervals. Tap-sound asynchrony was computed by comparing the onset of each tap with the onset of the sound to which it was closest in time. Sound onset times were subtracted from tap onset times, such that negative numbers correspond to anticipations of the pacing stimulus. “Variability” and “Asynchrony” scores were created by averaging across both rates (500 and 667 ms ISI) for the two variables.

**Table 3:** Dependent measures of tapping performance.

Tapping variability	The ability to tap consistently to a pacing stimulus; variability was calculated by computing the standard deviation of the inter-tap intervals. (Variability is the inverse of consistency).
Tap-sound asynchrony	The ability to align taps to a pacing stimulus in time; tap-sound asynchrony was computed by comparing the onset of each tap with the onset of the sound to which it was closest in time. Sound onset times were subtracted from tap onset times, such that negative numbers correspond to anticipations of the pacing stimulus. (Others refer to this measure as “anticipation tendency”).

### Statistical analyses

To analyze maturational changes of tapping performance cross-sectionally, Univariate Analyses of Co-Variance were performed with Non-Verbal IQ as the covariate, age group as the fixed factor and tapping performance (variability or asynchrony) as the dependent variable. To explore a potential association between musical experience and tapping variability and asynchrony, analyses were performed using musical experience as a categorical and a continuous variable. First, subjects were categorized as having musical experience or no musical experience, and then compared on tapping performance through a Univariate Analysis of Co-Variance using Non-Verbal IQ as a covariate, age group and musical experience as the fixed factors and tapping performance (variability or asynchrony) as the dependent variable. To understand the effect of music experience irrespective of subject categorizations, proportion of life spent playing music was calculated (years spent playing music divided by years of life), and then used as a predictor of tapping performance in multiple regression analyses.

## Results

### Age comparisons: variability and asynchrony

#### Variability

Overall, there was a developmental effect of age on tapping variability; the lowest and highest ages on the spectrum (children and older adults) had the largest amount of tapping variability, while the middle adults had the least amount of tapping variability.

**Fig 2:**
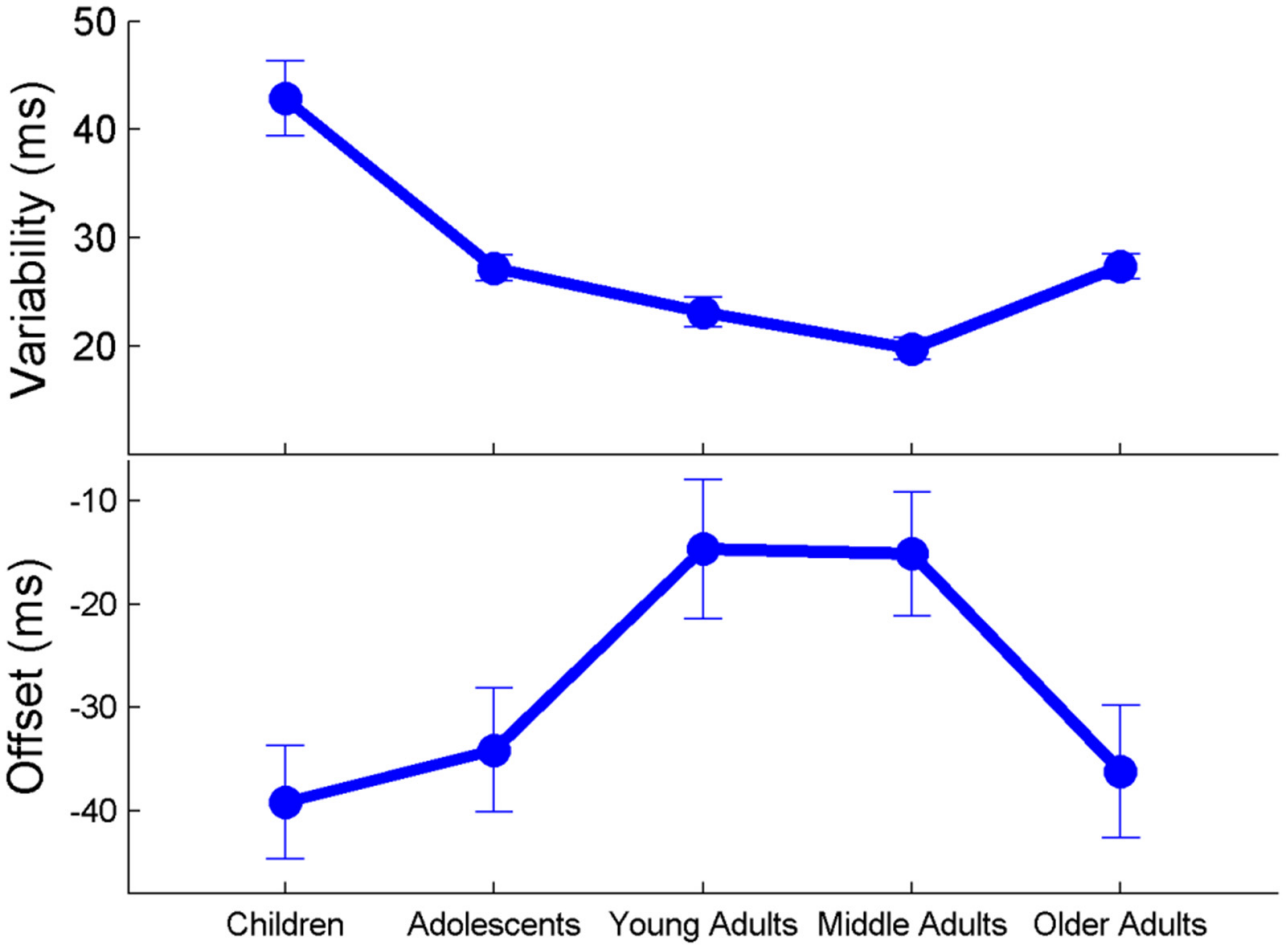
Beat Synchronization Changes Throughout Life. (A) The ability to tap to a beat improves with age into middle adulthood (ages 22 to 42.9), and then declines in older age, as assessed by tapping variability. (B) Anticipation of the beat was least accurate for children and older adults, as assessed by asynchrony. Error bars represent one standard error of the mean.

**Fig 3:**
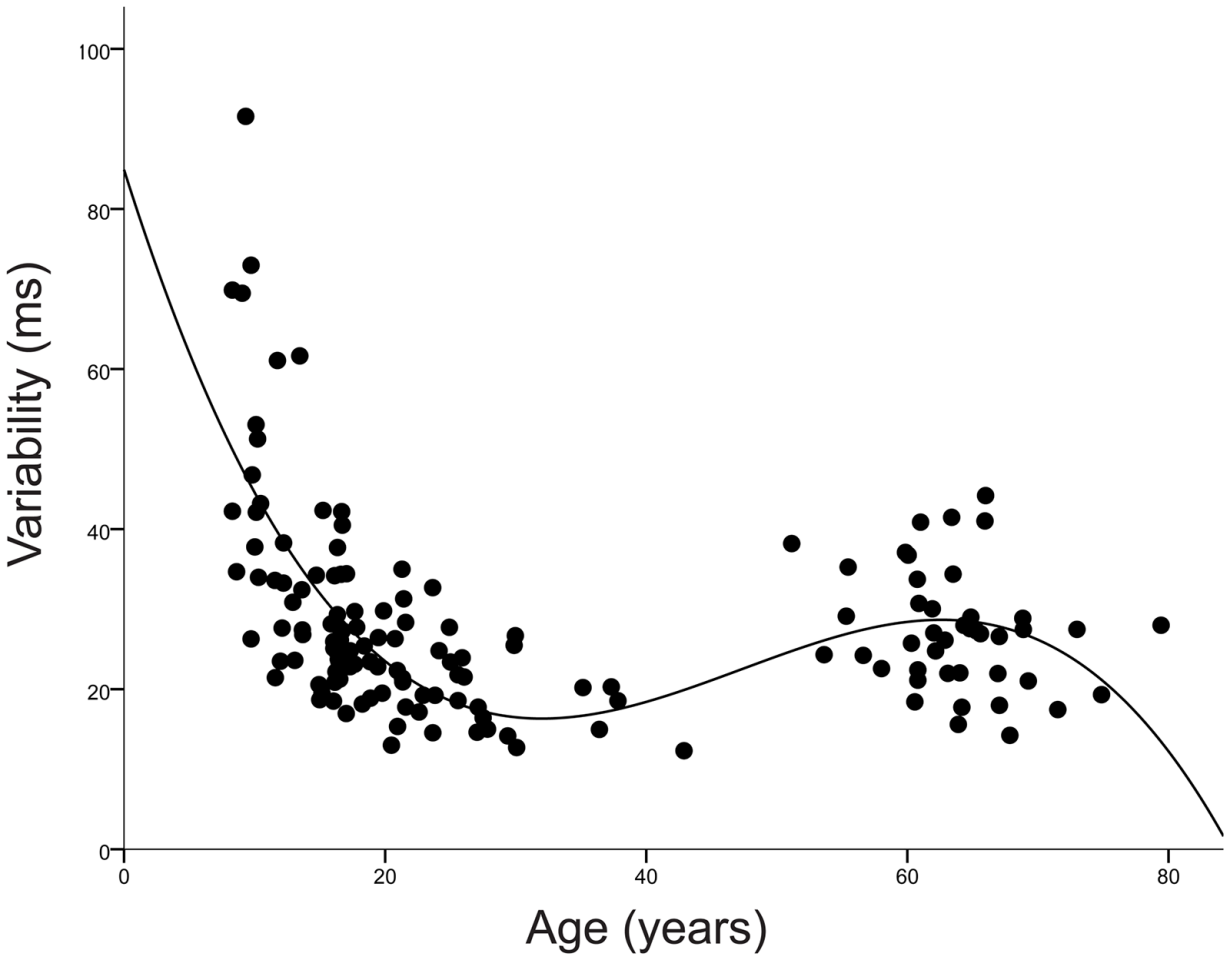
Non-Linear Development of Beat Synchronization Throughout Life. A cubic model fit the data to describe age-related changes in tapping variability, accounting for 42.6% of variance in tapping performance (Y = 85.055 + -5.192*X + .123*X^2^ +-.001*X^3^).

Average tapping variability across the lifespan is displayed in Fig 2a. A Univariate Analysis of Co-Variance (using Non-Verbal IQ as the covariate, age group as the fixed factor and tapping variability as the dependent variable) revealed that tapping variability differed among age groups (main effect of age group (F(4,141) = 21.58 *p* < .001). Bonferroni post-hoc tests indicated the children had significantly larger tapping variability in comparison to each age group (adolescents (*p* < .001), young adults (*p* < .001), middle age adults (*p* < .001) and older adults (*p* < .001)). There were no group differences in tapping variability between the adolescents and young adults (*p* > .2), however there were group differences between the middle adults and all other age groups (adolescents (*p* = .001), young adults (*p* = .049), and older adults (p = .035)). The older adults’ tapping variability was significantly larger than each age group except for the adolescents (young adults (p = .029), middle age adults (*p* = .035), adolescents (*p* > .4)). Overall, we found a cubic model fit the data to describe age-related changes in tapping variability (see Fig 3), accounting for 42.6% of variance in tapping performance (F(3,142) = 34.71, *p* < .001; Y = 84.90 + -5.166*X + .122*X^2^ +-.001*X^3^).

#### Asynchrony

Overall, there was a developmental effect of age on tap-sound asynchrony, in which the lowest and highest ages on the spectrum (children and older adults) tapped, on average, the furthest away from the beat. In other words, the children and older adults tapped far away from the beat (high amounts of asynchrony), whereas the middle age groups tapped close to the beat (low amounts of asynchrony).

**Fig 4:**
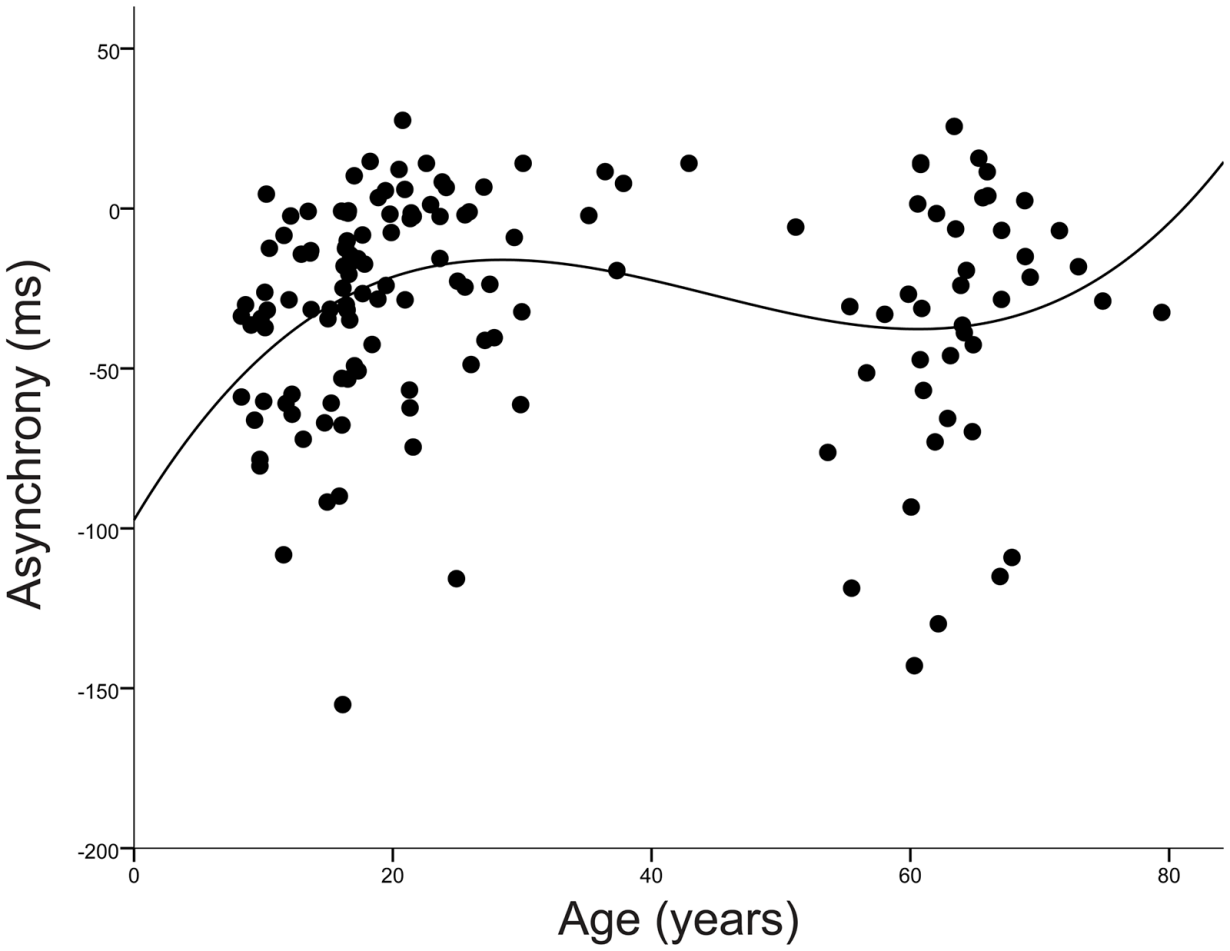
Non-Linear Development of Beat Synchronization Throughout Life. A cubic model fit the data to describe age-related changes in tapping asynchrony, accounting for 7.2% of variance in tapping performance (F(3,142) = 3.669, *p* = .014; Y = 97.375 + 6.77*X +-.175*X^2^ + .001*X^3^).

Tap-sound asynchrony across the lifespan is displayed in Fig 2b. A Univariate Analysis of Co-Variance (using Non-Verbal IQ as the covariate, age group as the fixed factor and tapping asynchrony as the dependent variable) revealed that tap-sound asynchrony differed among age groups (main effect of age group (F(4,141) = 3.01 *p* = .02). Bonferroni post-hoc tests indicated the children had significantly larger tap-sound asynchrony in comparison to young adults (*p* < .01) and middle age adults (*p* < .01). However, there were no differences between children and adolescents (p > .5) or older adults (*p* > .6). There were no group differences in tapping asynchrony between the adolescents and young adults (*p* > .1) or middle age adults (*p* > .05), nor were there group differences between the young adults and middle age adults (*p* > .9). The older adults had a significantly larger tapping asynchrony than each age group except the children and adolescents (children (*p* > .6), adolescents (*p* > .4), young adults (p = .047), middle age adults (*p* = .026)). Overall, we found a cubic model fit the data to describe age-related changes in tapping asynchrony (see Fig 4) accounting for 7.2% of variance in tapping performance (F(3,142) = 3.669, *p* = .014; Y = 97.375 + 6.77*X +-.175*X^2^ + .001*X^3^).

### Musical experience comparisons

#### Variability

Overall, individuals with musical experience had the least amount of tapping variability. Also, greater proportion of life spent playing music correlated with lower tapping variability.

**Fig 5:**
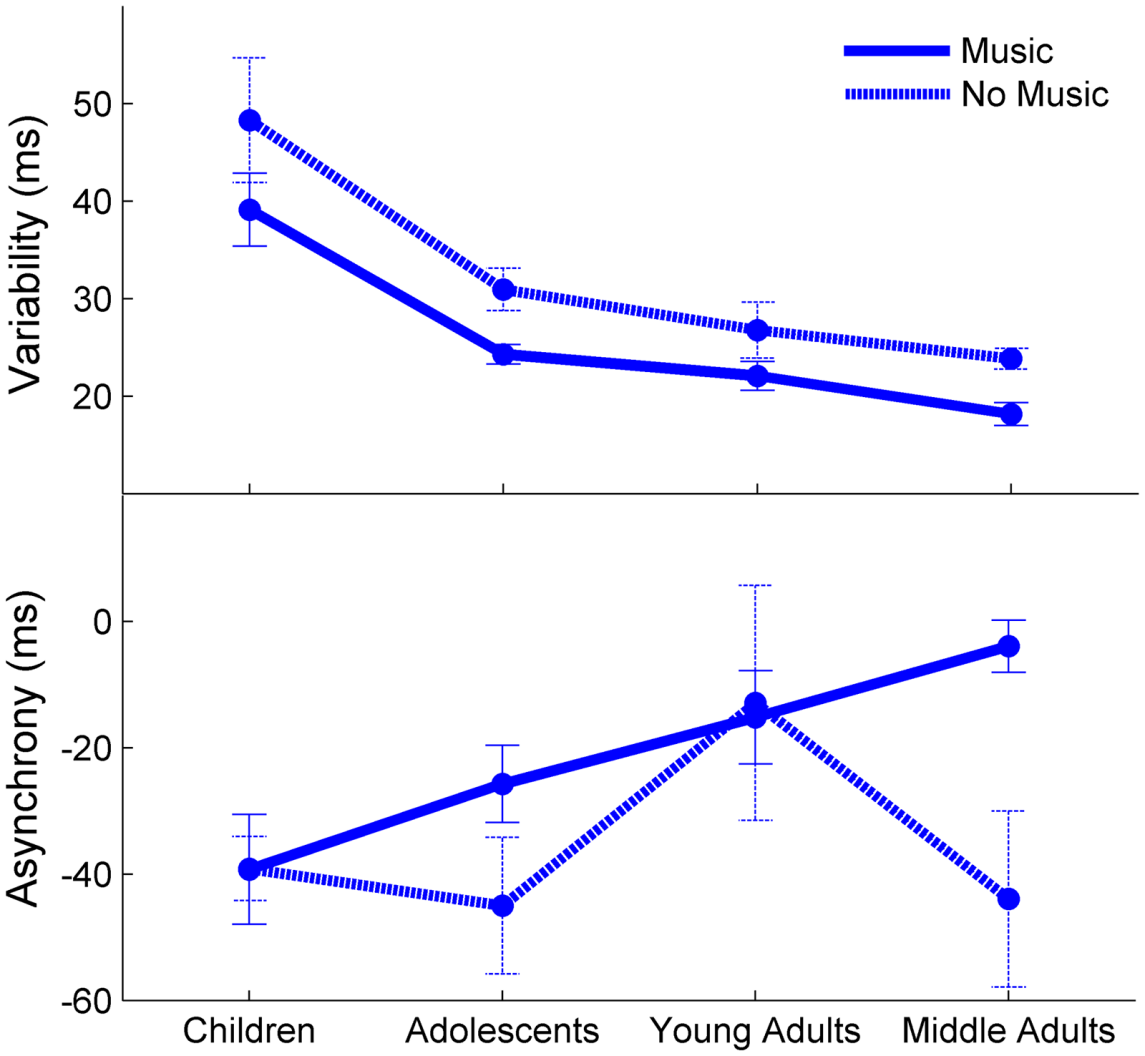
The Relationship Between Beat Synchronization and Music Experience. Individuals with musical experience perform better on a tapping task in comparison to individuals without musical experience, as assessed by two measures of tapping performance: (A) variability and (B) asynchrony. Error bars represent one standard error of the mean.

Average tapping variability across the four age groups with respect to musical experience is displayed in Fig 5. Older adults were excluded from this analysis because they did not report their musical history. A Univariate Analysis of Co-Variance (using Non-Verbal IQ as a covariate, age group and musical experience as fixed factors, and tapping variability as the dependent variable) revealed the developmental trajectories to be consistent with those found in the age group comparisons, indicated by a main effect of age group (F(2,100) = 21.901, *p* < .001). There was also a significant main effect of musical experience; performance was less variable in individuals with musical experience irrespective of age group (F(1,101) = 7.698, *p* = .007). Post-hoc t-tests revealed no within age-group difference for musical experience in the children (*p* > .1), nor young adults, (p > .1) yet a significant difference in the adolescents (*p* = .012) and middle age adults (*p* = .01). There was no age group by musical experience interaction, indicating musical experience did not alter the developmental trajectory of tapping performance (*p* > .8).

A Pearson’s correlation reveals a greater proportion of life spent playing music is significantly correlated with lower tapping variability (R(100) = -.255, *p* = .011). A multiple regression analysis used to predict tapping variability revealed over and above age, sex and Non-Verbal IQ, proportion of life spent playing music marginally reduced the variance of the model (ΔR^2^ = .025, F(1,95) = 3.875, *p* = .052, total R^2^ = .387, F(4,99) = 14.979, *p* < .001; β_music_ = -.165., *p* = .052).

#### Asynchrony

Overall, individuals with musical experience were tapped more closely to the presentation of the sounds (low mean tap asynchrony). Also, proportion of life spent playing music correlated with less mean tap asynchrony.

Mean tapping asynchrony across the four age groups with respect to musical experience is displayed in Fig 5. Older adults were excluded from this analysis because none reported musical experience. A Univariate Analysis of Co-Variance (using Non-Verbal IQ as a covariate, age group and musical experience as fixed factors, and tapping asynchrony as the dependent variable) revealed the developmental trajectories were generally consistent with those found in the age group comparisons, indicated by a trending main effect of age group (F(3,99) = 2.60, *p* = .057). There was a significant main effect of musical experience; tapping asynchrony was lower in individuals with musical experience irrespective of age group (F(1,101) = 4.55, *p* = .036). Post-hoc t-tests revealed no within age-group difference for musical experience in the children (*p* > .9), adolescents (p > .1) or young adults (p > .8), yet a significant difference in middle age adults (*p* = .001). There was no age group by musical experience interaction, indicating that musical experience did not alter the developmental trajectory of tapping asynchrony (*p* > .9).

A Pearson’s correlation reveals a greater proportion of life spent playing music is significantly correlated with lower mean tap asynchrony (R(100) = -.241, *p* = .016). A multiple regression analysis was performed to predict tapping mean asynchrony, revealing proportion of life spent playing music reduced the variance of the model, over and above age, sex and Non-Verbal IQ, (ΔR^2^ = .035, F(1,95) = 4.111, *p* = .045, total R^2^ = .193, F(4,99) = 5.694, *p* < .001; β_music_ = .195., *p* = .045).

## Discussion

We assessed paced synchronization to a beat across a wide age span and examined how having previous musical experience relates to performance. We found that the ability to move consistently to a beat improves across the lifespan into middle adulthood, yet in older adulthood, slightly declines. Also, there is a greater ability to closely align taps in time with beat presentation through adulthood, however this ability is less precise in older adulthood. We provide evidence that musical experience relates to lower tapping variability and lower mean tap asynchrony (beat alignment) during paced synchronization. These patterns align with previous research showing that the ability to tap to a beat continues to develop in childhood, becomes more variable with older age, and is enhanced with musical experience [16–18].

### Beat synchronization changes throughout life

Our results reveal that certain aspects of rhythmic entrainment, specifically tapping consistency and beat alignment (asynchrony), develop past childhood into adulthood. These two aspects are related, yet in certain cases may be mutually exclusive. For example, an individual could be highly synchronous (i.e. low variability) in their tapping performance, but their tapping occurs far away from the sound (e.g. they tap to the offbeats); this would be indicative of “high asynchrony”, and thus, they do not tap closely in time with the beat. Conversely, an individual with high variability (low synchrony) in their taps may have small asynchrony if they tap close to when the beat occurs in time. Our results suggest that tapping consistency has a developmental trajectory in which it improves through childhood to adulthood, reduce slightly in old age, and is smaller for individuals with music experience. Our results also suggest that tapping asynchrony is smaller for adults, yet is larger in childhood and older adulthood. Finally, tapping asynchrony is smaller for individuals with music experience, a finding consistent with previous studies showing musician’s tend to have little to no tapping asynchrony [32,33].

Several large cross-sectional studies have laid a strong foundation for understanding how rhythmic entrainment changes across the lifespan. For example, in a sample size of 305 individuals age 4 to 95 years old, perceptual-motor timing task performance varies with respect to age [17]. In a study of 286 participants, ages ranging from 8 to 88 years, synchronization improves during childhood into adulthood, where it is stable until older age [18]. Our results are consistent with these and others, revealing beat synchronization develops with age, that in older age, rhythmic entrainment is less stable, and finally, that individuals with musical experience have different beat synchronization profiles when compared to individuals without musical experience [16].

Many learned talents, such as playing an instrument or speaking a second language, have a “sensitive period” in which learning the skill early on is most beneficial, after which learning continues for a few years past this period yet plateaus in adolescence [34]. Our results suggest that rhythmic entrainment improves with age past adolescence and may not plateau until young or middle adulthood [16–18]. Though these learned talents are interactions between intrinsic maturation and unique life experiences, our results align with others [17] which propose the sensitive period of rhythmic entrainment may persist into older age groups than previously thought.

One possible explanation for this prolonged learning period is that integration across multiple sensory and motor networks is necessary for performing the multimodal finger-tapping task. Functional connectivity between auditory and motor areas continues to mature into young adulthood [35–37] and declines in older adulthood [38]. We speculate that beat synchronization may rely in part on the synchronous integration of multiple regions of the brain, which might also help explain the declines in rhythmic entrainment performance observed in the older adults in our study. However, additional factors may contribute to the relatively poor entrainment performance in older adults, as has been observed in previous studies and is replicated here. For instance, older adults have deficits in motor coordination, balance and gait, as well as movement slowing [39]. Moreover, aging is associated with decreased brain connectivity [38], degeneration of neurotransmitter systems [39], and a loss of synchronous firing throughout the auditory system [40–42]. This loss in neural synchrony may be a key contributor to the older adults’ reduced ability to entrain to a beat, especially given the established link between tapping performance and subcortical neural synchrony [26].

With evidence in mind showing a significant link between tapping variability and IQ, it may be possible that in this study, synchronization performance is mediated by intelligence [43]. However, in the regression analyses, IQ did not significantly account for any variance in the models. Also, given the age-group differences in IQ from the outset, we treated IQ as a covariate in our analyses, finding that when controlling for IQ, there was still an effect of age. Future research with a wide range of age and IQ could explore developmental effects of IQ on beat synchronization more fully.

### Beat synchronization, music experience, & implications for everyday communication

These results provide evidence for links between musical experience and certain aspects of tapping performance, specifically variability and beat alignment. We show, though a marginal effect, that individuals with more music experience are less variable tappers. This pattern of results is intuitive, since precise rhythm production is a key component of musical performance, and also aligns well with previous work that has shown links between musical experience and greater tapping abilities [16,19,20]. However, our cross-sectional design does not allow us to determine whether these group differences are a product of training or a pre-existing difference. Future work, such as longitudinal studies with random music training assignment, should investigate whether the relation we found between musical experience and synchronization ability reflects an effect of training or the influence of pre-existing differences. For example, individual differences in musical aptitude, cognition, and personality have been found between individuals who seek music training compared to those who do not [24,44,45].

The positive relationship between years of music experience and beat keeping skills suggests that the putative musician enhancement we report is due, at least in part, to training. If, in fact, musical training can enhance auditory-motor synchronization, this possibility may have consequences for the field of language development. Musical training is associated with superior linguistic abilities such as reading [46,47] as well as auditory skills [as reviewed in 21], and thus, rhythm may be one of the primary factors driving musical training’s benefit for language. For example, reading performance, whether impaired or across a continuum of normal reading performance, relates to the ability move to a beat [25,48–50]. Moreover, phonological abilities improve with rhythmic training [51]. Our results may also be of interest to the study of literacy given evidence for correlations between reading aptitude and tapping variability; by providing a description of changes in tapping variability across the lifespan our results may be of interest to scientists developing rhythmic interventions for language and literacy [51].

### Future directions

Our participant population did not include any older adults with musical training. As a result we cannot say with certainty whether or not the musical experience correlation with beat synchronization performance is maintained into older age. Given that older adult musicians have superior auditory cognitive skills and more synchronous neural responses to sound than their age-matched non-musical experience peers [23,52], we predict that older musicians would also have increased beat synchronization performance. Furthermore, the musician effects we see cannot necessarily be fully accounted for by the training itself, and may instead reflect pre-existing differences. Controlled studies, both cross-sectional and longitudinal, are required across the lifespan to determine whether and how musical training may influence beat synchronization abilities.
